# The combined cartilage growth – calcification patterns in the wing‐fins of Rajidae (Chondrichthyes): A divergent model from endochondral ossification of tetrapods

**DOI:** 10.1002/jemt.24217

**Published:** 2022-08-03

**Authors:** Ugo E. Pazzaglia, Marcella Reguzzoni, Renata Manconi, Piero Antonio Zecca, Guido Zarattini, Monica Campagnolo, Mario Raspanti

**Affiliations:** ^1^ DSMC University of Brescia Brescia Italy; ^2^ DMC University of Insubria Varese Italy; ^3^ DVM (Zoology Lab) University of Sassari Sassari Italy; ^4^ DBSV University of Insubria Varese Italy

**Keywords:** calcified cartilage, chondrocytes, mineralization patterns, radials, Rajidae wing‐fins, skeletal growth

## Abstract

The relationship between cartilage growth – mineralization patterns were studied in adult Rajidae with X‐ray morphology/morphometry, undecalcified resin‐embedded, heat‐deproteinated histology and scanning electron microscopy. Morphometry of the wing‐fins, nine central rays of the youngest and oldest specimens documented a significant decrement of radials mean length between inner, middle and outer zones, but without a regular progression along the ray. This suggests that single radial length growth is regulated in such a way to align inter‐radial joints parallel to the wing metapterygia curvature. Trans‐illumination and heat‐deproteination techniques showed polygonal and cylindrical morphotypes of tesserae, whose aligned pattern ranged from mono‐columnar, bi‐columnar, and multi‐columnar up to the crustal‐like layout. Histology of tessellated cartilage allowed to identify of zones of the incoming mineral deposition characterized by enhanced duplication rate of chondrocytes with the formation of isogenic groups, whose morphology and topography suggested a relationship with the impending formation of the radials calcified column. The morphotype and layout of radial tesserae were related to mechanical demands (stiffening) and the size/mass of the radial cartilage body. The cartilage calcification pattern of the batoids model shares several morphological features with tetrapods' endochondral ossification, that is, (chondrocytes' high duplication rate, alignment in rows, increased volume of chondrocyte lacunae), but without the typical geometry of the metaphyseal growth plates.

**Research Highlights:**

1. The wing‐fins system consists of stiff radials, mobile inter‐radial joints and a flat inter‐radial membrane adapted to the mechanical demand of wing wave movement.

2. Growth occurs by forming a mixed calcified‐uncalcified cartilage texture, developing intrinsic tensional stresses documented by morphoanatomical data.

## Graphical abstract

(a) Bright field and (b) Phase‐contrast image. The cartilage growth pattern forms a mixed texture of calcified‐uncalcified matrix (not undergoing remodeling) which develops intrinsic, tensional stresses inside the tissue, histologically documented by elongation of chondrocyte lacunae and collagen fibers in the resilient uncalcified zones and by fractures in the neighboring calcified matrix.

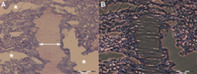

## 1 INTRODUCTION

The cartilage plays a fundamental role in the skeletal development of vertebrates; in mammalians, this is then replaced by bone through endochondral ossification (Hall, 2005). Characteristic transformations (such as “hypertrophy”) occur in chondrocytes whose surrounding matrix is undergoing to mineral deposition, as widely investigated in the literature on bio‐mineralization (Bonucci, 1971; Pazzaglia et al., 2016, 2018).

In endochondral ossification, cartilage is a temporary tissue before it is replaced by bone after the foetal and the metaphyseal growth phases, remaining later only on the surface of the joints during the whole lifetime. Otherwise, from tetrapods and birds, the cartilage of Chondrichthyes forms the definitive skeleton segments, which do not undergo secondary substitution (remodeling). The endoskeleton and fins cartilage of the latter has drawn deep interest in their phylogenesis (Compagno, 1973, 1977; Maisey, 1984) because it represents a distinctive and unique model of cartilage growth associated with mineral deposition diverging from the pattern observed in most living vertebrates and the fish class of Actinopterygii. Batoids developed wing‐like structures by fusing the pectoral fins used to swim. The swinging movement of the waves across them during steady swimming gives this movement power (Heine, 1992; Klausewitz, 1964; Rosenberger, 2001). The wing‐fins skeleton consists of a series of parallel rays formed by serially aligned radials linked by amphiarthroses with a limited range of movement and restrained laterally by a thick inter‐radial membrane (Pazzaglia et al., 2022; Schaefer & Summers, 2005). The radials of the first median line articulate with the pterygia (propterygia, mesopterygia, neopterygia, and metapterygia). Other, shorter rays articulate with the pelvic girdle basipterygia (Compagno, 1999). Overall, the calcified cartilage segments of wings form a functional and mechanical flat system of variable stiffness/flexibility that can translate the contraction of the powerful dorsal and ventral wing muscles into the undulatory‐oscillatory locomotion of these fish species (Rosenberger, 2001).

The Rajidae skeletal morphology offered a cartilage model stiffened by mineral deposition and adapted to the mechanical demand of their motion. Basic units, that is, tesserae, form this composite tissue of calcified and uncalcified matrix.

This study has been carried out in the species *Raja clavata* with the following purposes: 1) to analyze with X‐rays and trans‐illumination morphometry the wing‐fins radials growth pattern; 2) to compare the calcified layout of the wing‐fins, the pelvic girdle basipterigium fins radials and the tessellated endoskeleton segments; 3) we used a combination of light, scanning electron microscopy (SEM) and heat deproteination to study the mineral deposition progression of cartilage scaffold.

## 2 MATERIALS & METHODS

### 2.1 Experimental animals

The study was carried out on five adult specimens of *Raja clavata* Linnaeus, 1758, family Rajidae, class Chondrichthyes (Serena et al., 2010) captured in the Western Mediterranean and purchased from commercial sources (Mercato Ittico di Milano and Mercato Ittico di Sassari, Italy). Animal welfare laws, guidelines and policies were not applicable.

The fresh fishes were weighed and measured fresh before dissection (Table 1). Wing‐fins of specimens 1 and 5 were used for morphometry, while those of specimens 2, 3, and 4 were dissected and processed for histology, heat deproteination and SEM study.

**TABLE 1:** Sex, weight, disk‐width, total‐length and ratio disk‐width/total‐length of the 5 studied specimens of *Raja clavata* (Chondrichthyes).

Specimen	Sex	Weight (gm)	Disk‐ width (cm)	Total ‐ length (cm)	Ratio d‐w/t‐l
1	F	110	19	30	0.63
2	F	420	26	39	0.67
3	F	510	30	43	0.69
4	M	930	35	56	0.62
5	F	1520	37	60	0.62

### 2.2 X‐Rays morphometry and trans‐illumination imaging

**FIGURE 1:**
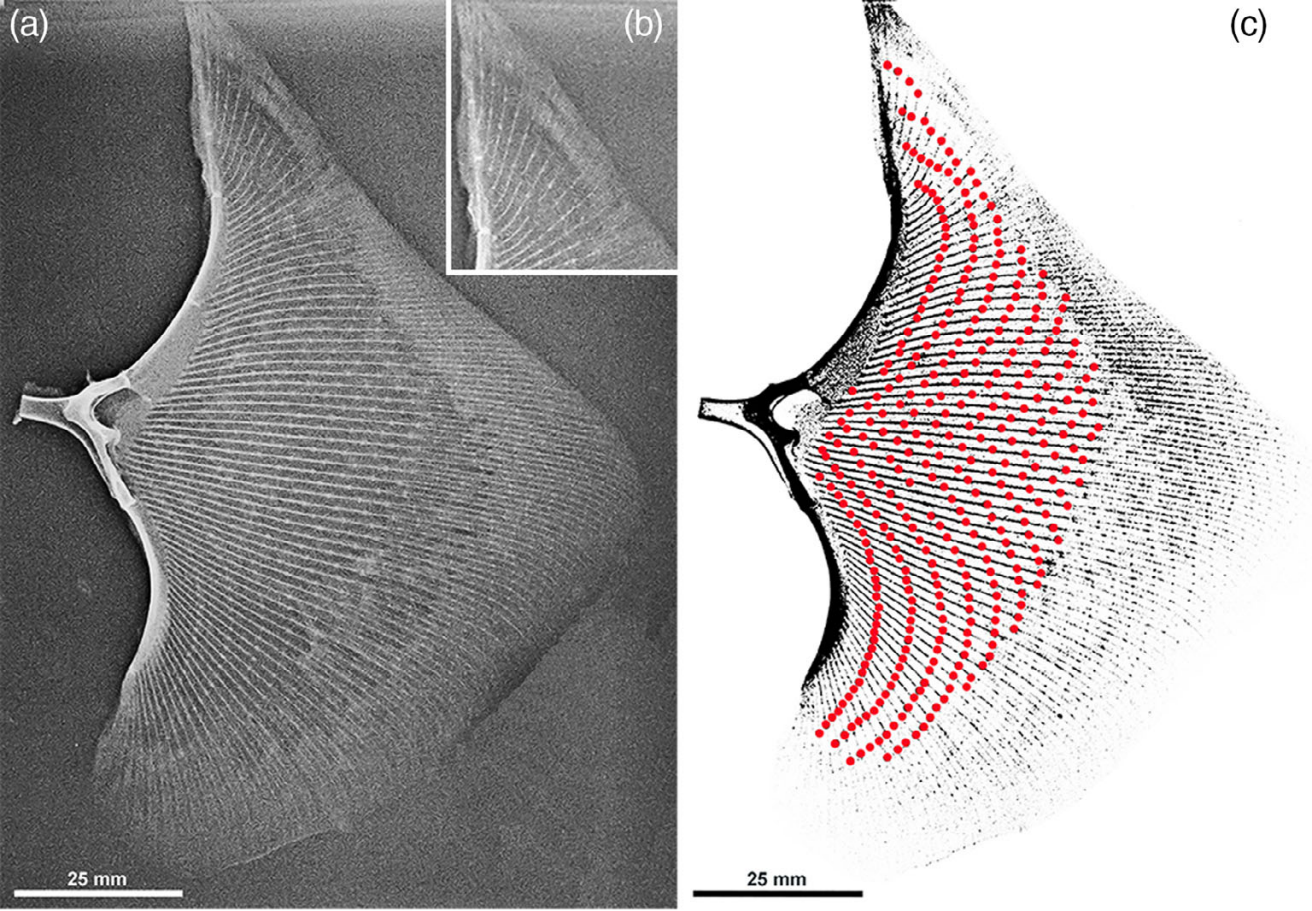
*Raja clavata* (Chondrichthyes) X‐ray (A‐P projection) of the right wing‐fin of specimen 5. (a) Wing‐fin whole image shows the layout of rays radials and joints with propterygium, mesopterygium, neopterygium, and metapterygium. The central sector rays are oriented along a straight line perpendicular to the central pterygia. (b) Detail of the anterior wing‐tip showing the most anterior rays parallel to the spine axis and the low number of radials in the ray. (c) Pattern of inter‐radial joints (enhanced by red spots) with a parallel alignment to the propterygia curvature.

X‐rays of each specimen's right and left‐wing fins and pelvic girdle basipterygia were taken in the dorsoventral projection with Siemens equipment (Siemens AG, Munich, Germany). The number of rays and radials along each ray was determined in wing‐fins X‐rays, while for the pelvic basipterygia, only the number of rays was determined. In the right wing‐fin of the youngest and oldest specimen (weight 110 and 1520 g, respectively), the rays of the central sector, whose first radials line articulated with the mesopterygium and neopterygium (Compagno, 1999), were divided with two parallel cuts from the basal pterygia to the outer border of the wing. The skin and muscles on the dorsal and ventral surface were gently dissected with a scalpel (leaving only the inter‐radials membrane and skin layer of ≈2.5 cm on the wing outermost border) to avoid damaging the most external, very thin radials. High‐definition radiograph images of the calcified segments were obtained with a NewTom CT (New Tom, Verona, Italy) for radials length measurements. The same calcified segments (plunged in Petri capsules in a glycerol solution bath) provided the trans‐illumination images of radials at magnifications from 1.25 to 40x with a stereo microscope Olympus SXZ 7 and an Olympus BX 51 (Olympus Ltd, Tokyo, Japan). The selected central sector included nine rays, corresponding to the longest rays of the fin. The sketchy mesopterygium and neopterygium 1st segment of radial (characterized by high variability of the distance between propterygia border‐1st radial joint, that is, line 0) was excluded from statistics. Trans‐illumination images of fixed and unembedded specimens (without skin and radial muscles) of radial series were obtained through observation in a large Petri capsule filled with glycerol solution. The central sectors of specimens 1 and 5 were selected for morphometry based on key morphotraits: 1) the longest, straight rays of the wing with the higher number of radials; 2) a regular line of bifurcating radials (line 9) occurring at ≈2/3rd of the whole length, with 8 radial lines interposed between line 0 and 9 (bifurcation line) and approximately more than 8 lines externally. It was not possible to assess with precision the number of the outer calcified segments due to their very‐thin diameter (the ventral and dorsal skin layer with dermal denticles was left to avoid damage with dissection of the inner tissue texture); 3) the medial joints between meso/neopterygia–1st radials were not definable. Therefore the latter were excluded from measurements (line 0); 4) the 8 proximal radials lines below bifurcation was divided into two zones **1a**, **1b (**1 = youngest specimen) and **5b**, **5b** (5 = oldest specimen), corresponding respectively to radials lines 1–4 and 5–8, while the external zones above bifurcation **1c**, **5c** corresponded to radials lines 10–13 (Figure 1d,e). The bifurcating radials of line nine were not measurable. However, the latter's identification accuracy was high through the criterion of a single inter‐radial joint medially and two distinct joints laterally.

This experiment analyzed high‐definition digitalized images with the program Cells (Soft Imaging System GmbH, Munster, Germany). The statistical analysis was carried out with the MedCalc program (MedCalc Software Ltd, Ostend, Belgium) using the Student *t*‐test to compare the radial mean length difference between zones **1a ‐ 1b** ‐ **1c, 5a ‐ 5b** ‐ **5c** and **1a ‐ 5a, 1b ‐ 5b, 1c** ‐ **5c**. A probability of *p* < .05 was considered statistically significant. Two investigators obtained repeated length measurements independently (MR and GZ). The difference in the mean analysis (Bland & Altman, 2010) was applied to these data sets. The difference of each paired measurement (intra‐observer, repeated after 30 days, and inter‐observers) was plotted against differences of the observers whose only source of variability was the measurement error. The differences between the inter‐observer and intra‐observer data sets had a degree of agreement >95% confidence interval for both.

Trans‐illumination allowed highlighting of the “tiles” calcified texture and layout of radials and pterygia.

### 2.3 Heat Deproteination

Dry specimens (1 × 0.5 cm) of the wing‐fins radials and 1 mm thick transverse sections of the pterygia lying on glass slides were subjected to heat deproteination in a muffle furnace at 400°C for 24 h, then mounted with a cover slip and observed at higher magnification (40–200x) with an Olympus BX51 microscope (Olympus Optical Co LTD, Japan).

### 2.4 Histology

1). Specimens of radials and pterygia were decalcified in 10% ethylene‐diamine tetra‐acetic acid solution for 30 days and were embedded in paraffin blocks. Sections were stained with hematoxylin–eosin and mounted on glass slides. 2). Undecalcified specimens were embedded in Technovit 7200 resin (Kulzer GmbH, Germany), and 150–200 μm thick sections were prepared with the Exact cutting/grinding system (Exact Advanced Technology GmbH, Norderstedt, Germany) and stained with methylene blue‐acid fuchsine. 3). Single, heat‐deproteinated “tiles” were mounted on glass slides and observed under the light microscope.

### 2.5 Scanning electron microscopy

Longitudinally sectioned surfaces of radials were dehydrated in increasing concentration of ethanol solutions, CO_2_critical point dried and secured on stabs with a bi‐adhesive tape. They were coated with a thin layer of gold in a vacuum sputter‐coater (Emitech) and examined with a Philips XL30 scanning electron microscope using secondary electron imaging (SEI) and backscattered electron imaging at 20 kV and 10 mm of working distance.

## 3 RESULTS

### 3.1 X‐rays

The wing‐fins of the studied Rajiae were symmetric, showing an approximate triangular outline with two rounded lateral and posterior angles. The anterior wing skeleton ends with the sharpened propterygium tip, as shown by X‐rays, but not evident from the fish's outer look because the skin joins the head and rostrum together with the wings.

**FIGURE 2:**
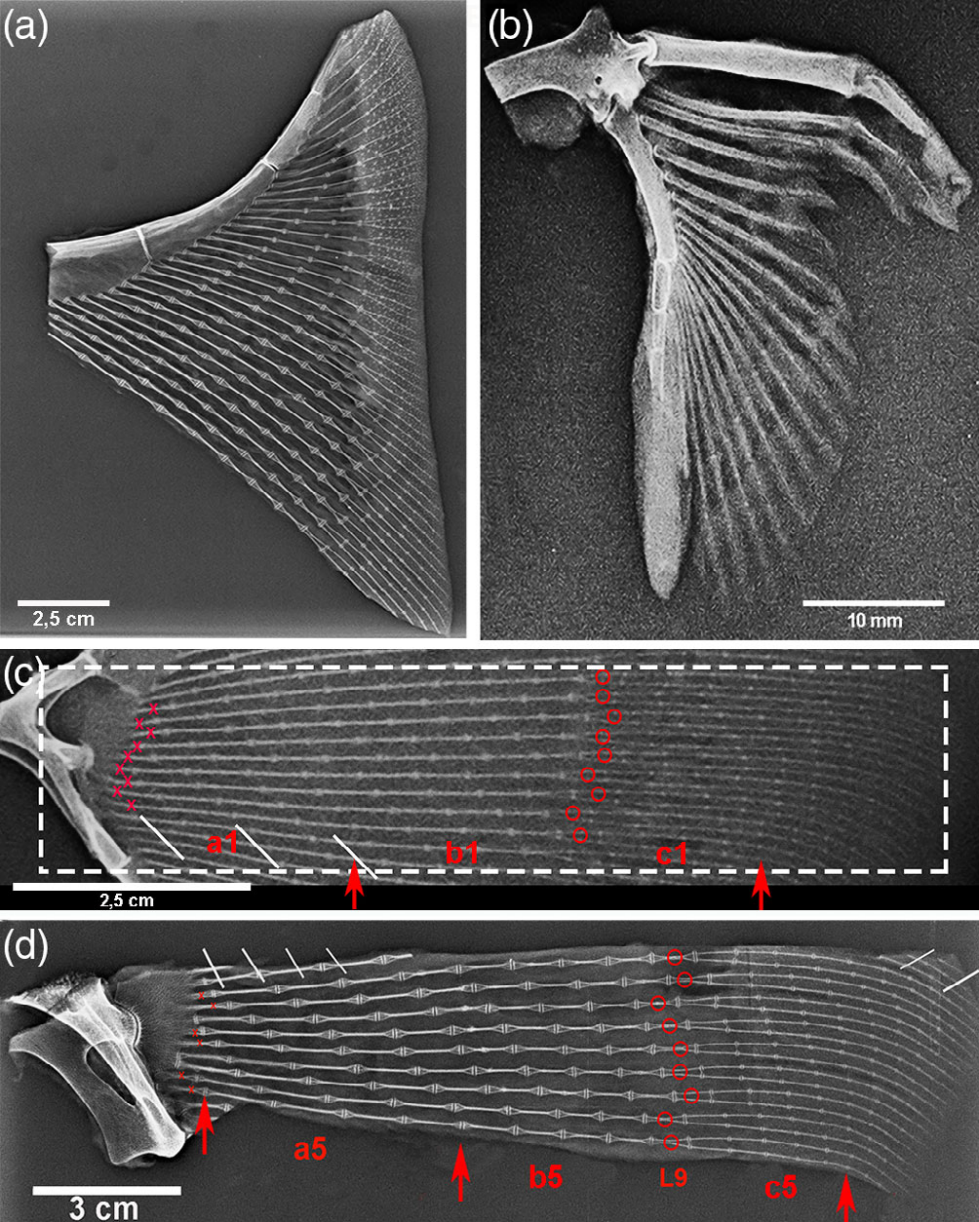
*Raja clavata* (Chondrichthyes) X‐rays of specimens 1 & 5. (a) Specimen 5, high‐resolution X‐rays of the propterygium wing‐fin anterior sector (after dissection of skin and muscles) showing the increasing number of radials from the anterior tip towards the central sector and the joint angles. In the outer band (with skin not dissected), the dermal denticles are superimposed on the thin, most external radials. (b) Specimen 5, X‐rays of pelvic basipterygium with the 1st line of long and thick radials. The thicker compound radial forms a diarthrodial joint with the pelvic girdle. (c–d) X‐rays of the 9 central sectors rays of specimens 1 and 5 with the labeled references for morphometry.

We have counted 68 to70 rays in the wing fins of the studied specimens. This slight variation is explained by superimposition in X‐rays of the most anterior and posterior rays with the tip of the propterygium and the metapterygium. The single, total ray length and the number of radials in the ray increase progressively from the anterior extremity to the central sector to decrease in the posterior, rounded angle of the wing. The series of rays articulating with the mesopterygium and neopterygium (central sector of the wing‐fin) is the longest, with a higher number of radials and a perpendicular direction to the fish's long axis (Figure 1a,b). The length of the radials calcified axis decreases along the ray from pterygia to the outer border, doubling the column of aligned central tiles (ACT) at ≈2/3rd of the whole length, which becomes progressively thinner towards the ray tip (Figure 2a). The segmentation through inter‐radials joints is maintained outside the bifurcation line 9. An ordered layout of the inter‐radial joints can be observed in the whole wing‐fins X‐rays where the joints draw below the bifurcation line 9 in two series of arches: the first (anterior) running parallel to the propterygium curvature, the posterior to the metapterygium and intersecting at the level of wing central sector corresponding to the mesopterygium and neopterygium) (Figure 1c). The pelvic girdle basipterygia articulate with ≈20 shorter rays than the wing fins, with no bifurcation. The first line of radials is characterized by longer and thicker segments than those following outwardly along each ray. Also, their X‐ray mineralized texture appeared different from the wing‐fin radials (Figure 2b).

### 3.2 X‐rays morphometry

Statistical analysis of radials mean length measured on X‐rays of the right wing‐fin central sector (Figure 2c,d) documented a significant decrement among zones **a**, **b**, and **c** and between the corresponding zones **1a**, **1b**, **1c** and **5a**, **5b**, **5c** of the youngest and oldest specimen (Table 2), representing the somatic growth of the species *Raja clavata*. However, the radials length decrement along each ray in both the target specimens 1 and 5 was not regular, suggesting that the length growth of single radials could have been set in such a way to keep the curvature of the inter‐radials joints in the wing (Figure 1b). The most outer radial length of the ray was not assessable with X‐rays due to super‐imposition of skin and small dermal denticles (Figure 2a).

**TABLE 2:** *Raja clavata* (Chondrichthyes). Comparison of radials mean length of specimens 1 and 5 between right wing‐fin zones a, b, and c in the central sector 9 rays (radial line 0 and 9 excluded from measurements).

Central sector zones	*n*	Specimen 1 (110 g) length (mm) ± *SD*	n	Specimen 5 (1520 g) length (mm) ± *SD*	
*Radial line 0*					
Zone **a** Radial lines 1–4	36	10.75 ± 0.1	36	14.2 ± 0.8	*p* < .001
Zone **b** Radial lines 5–8	36	8.6 ± 0.9	36	9.9 ± 1.6	*p* < 0.001
*Radial line 9*					
Zone **c** Radial lines 10–13	72	5.2 ± 1.1	72	5.6 ± 1.1	*p* < 0.001

a1	Versus	a5	*p* = 0.002
**b**1	Versus	**b**5	*p* = 0.02
**c**1	Versus	**c**5	*p* = 0.65 (n s)

### 3.3 Microscopic trans‐illumination morphology

**FIGURE 3:**
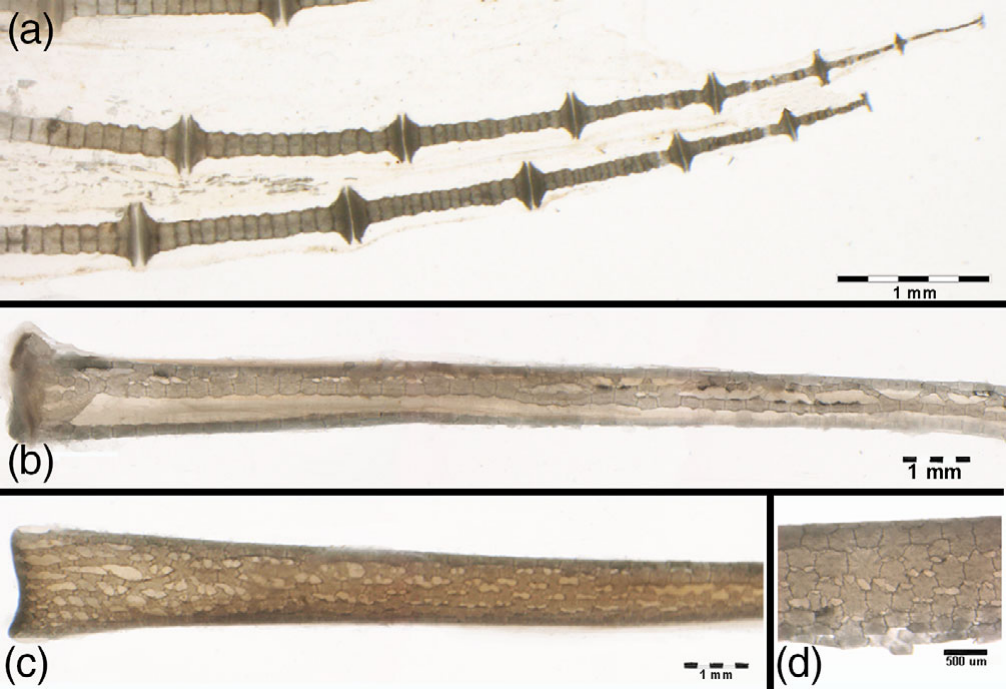
*Raja clavata* (Chondrichthyes) trans‐illumination images of wing‐fin and pelvic radials. (a) (1.25x) Wing‐fin radials (outer band) with the mono‐columnar morphotype of aligned, cylindrical tiles becoming progressively thinner towards the tip. (b) (5.6x) Pelvic basipterygium radials with multi‐columnar morphotype. (c) (5.6x) Pelvic basipterygium radials with tesseral shape modulation towards the crustal‐like morphotype. (d) (40x) Detail of the tiles transformation into the crustal‐like morphotype (typical of endoskeleton segments).

The trans‐illumination imaging allowed documenting the inner, mineralized texture of wing‐fin radials after dissection of skin and muscles. The typical pattern, either within or outside the bifurcation line 9, was that of cylindrical tiles laying one on top of the other to form a central calcified column and branching at the extremities to support the larger disks of the inter‐radials joint (Figure 3a).

**FIGURE 4:**
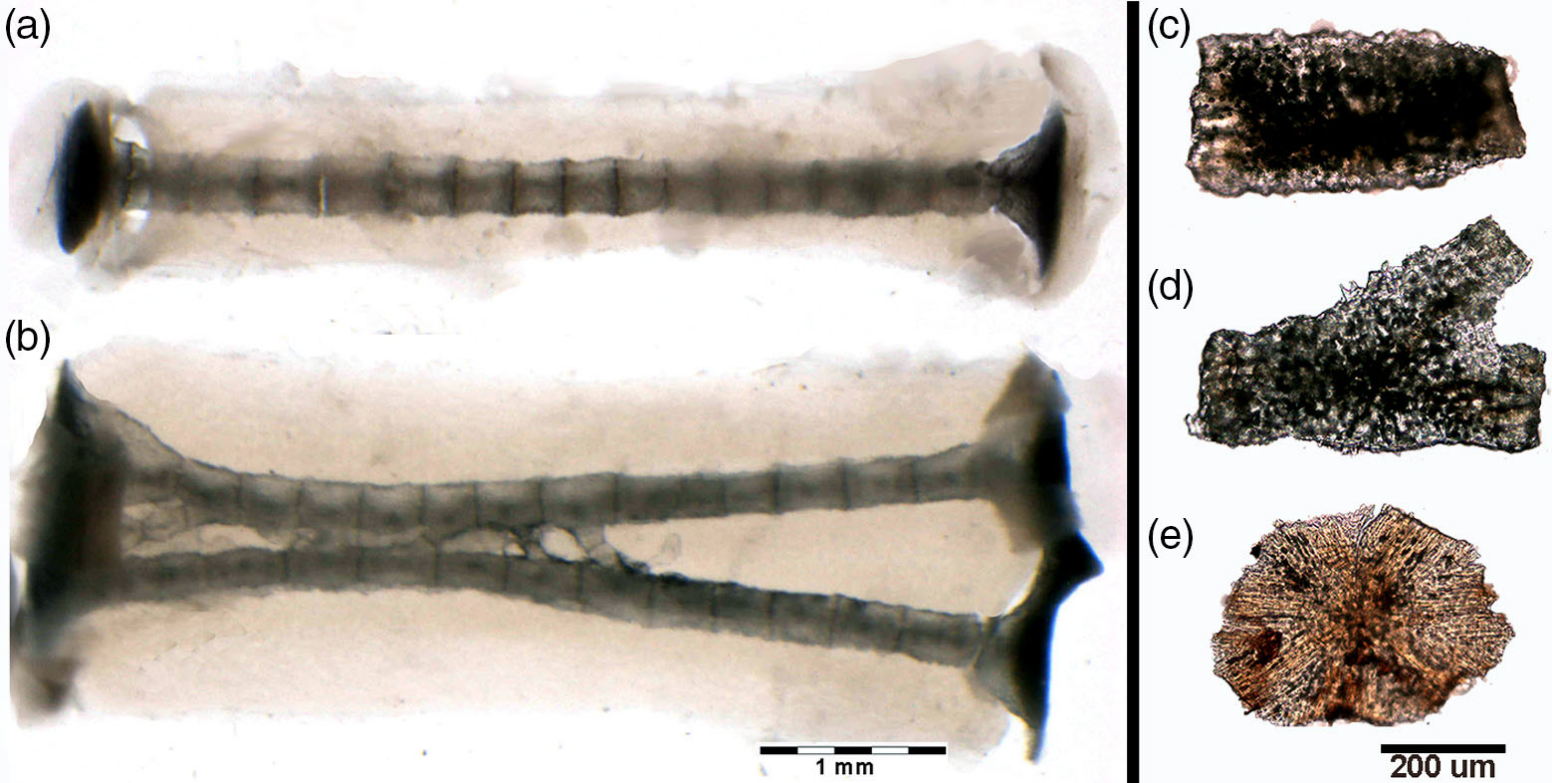
*Raja clavata* (Chondrichthyes) trans‐illumination images of mono‐columnar and bifurcating wing‐fin radials and heat‐deproteinated images of separated tesserae. (a–b) (2.5x) Wing‐fin mono‐columnar and bifurcation radial (line 9) showing the radial, uncalcified cartilage body and the central column formed by aligned tiles. (c–e) (100x) Morphotypes of heat‐deproteinated tiles: cylindrical (C), bifurcating (D) and polygonal (E). The black deposits inside the mineralized texture are carbon deposits of combustion.

The 1st line radials of the pelvic girdle basipterygia are longer and thicker than those of the wing‐fins, showing an inner, calcified texture formed by more columns of cylindric tiles with transverse interconnections (Figure 3b). A gradual transformation from the multi‐columnar pattern to a 3‐D reticular network was correlated to the increment of the radial diameter, the latter being associated with the variation of the tiles morphotype (Figure 3c,d). To sum up, briefly, four patterns of calcified tiles aggregation could be distinguished: 1) mono‐columnar; 2) bi‐columnar; 3) multi‐columnar, and 4) crustal‐like texture (Figure 4a,b).

### 3.4 Heat deproteination

**FIGURE 5:**
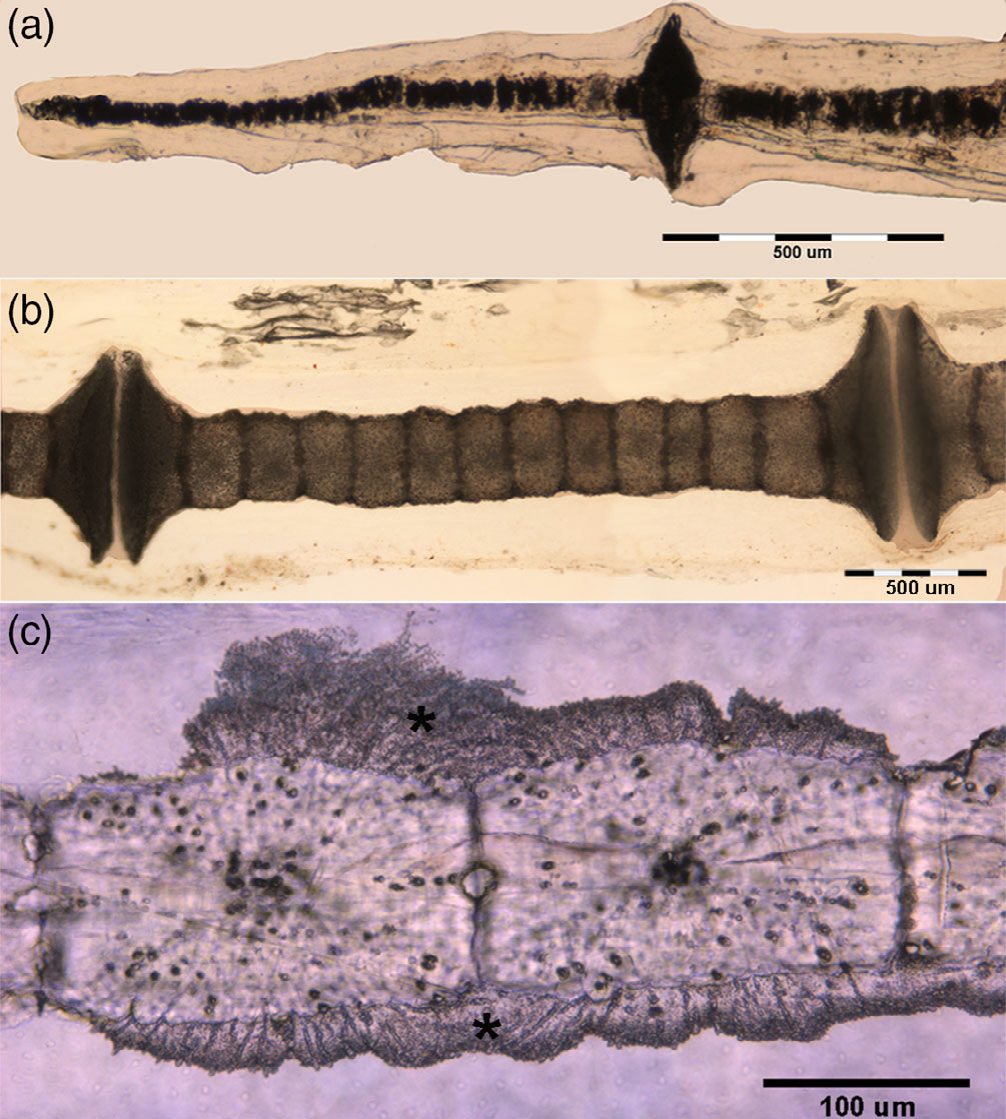
*Raja clavata* (Chondrichthyes). Heat‐deproteinated (400°C) images of wing‐fin radials at different ray levels. (a) (40x) Small, globular Ca_2_PO_4_ spherules of the radials apical segment mixed with black carbon deposits produced by combustion of the organic matrix. (b) (100x) Mono‐columnar radialshowing the calcified, cylindrical tiles: at the proximal and distal ends, the mineralized cartilage enlarges to form the disks of inter‐radial joints. (c) (200x) Detail of cylindrical tiles showing a separation plane between individual tiles. The black dots in the calcified tissue mass are carbon deposits into the chondrocyte lacunae. The compact calcified mass extending externally over several neighboring tesserae corresponds to a different type of mineralization which has also been reported around polygonal tesserae of the endoskeleton as “endophytic masses”.

Heating at 400°C burns the cartilage's organic phase, revealing the mineral component alone but leaving the segment's calcified structure intact (Pazzaglia et al., 2016). The heated samples are brittle, and the radials “tiles” units are easily separated. The residual, organic phase combustion products leave black sediments in fissures and chondrocyte lacunae of the calcified mass. Separated “tiles” showed three morphotypes: 1). Cylindrical; 2). Bifurcated (observed below the proximal and distal disk of inter‐radials joints or forming intercalary connections between multi‐columnar radials); 3). Polygonal (alike the “tesserae” of the endoskeleton) (Figure 4c–e). The fissures and globular cavities observed in heated specimens were filled in vivo by uncalcified cartilage matrix and chondrocytes. Mineralized septa bridged the “tiles” laid one on top of the other in the column (Figure 3a), while their perimeter decreased from the medial to the outer wing‐fin zone. The calcified column of the wing‐fin tip radials was formed by a single line of tiny spherules (Figure 5a), documenting the early phase of mineral deposition (Figure 5b,c).

### 3.5 Histology

**FIGURE 6:**
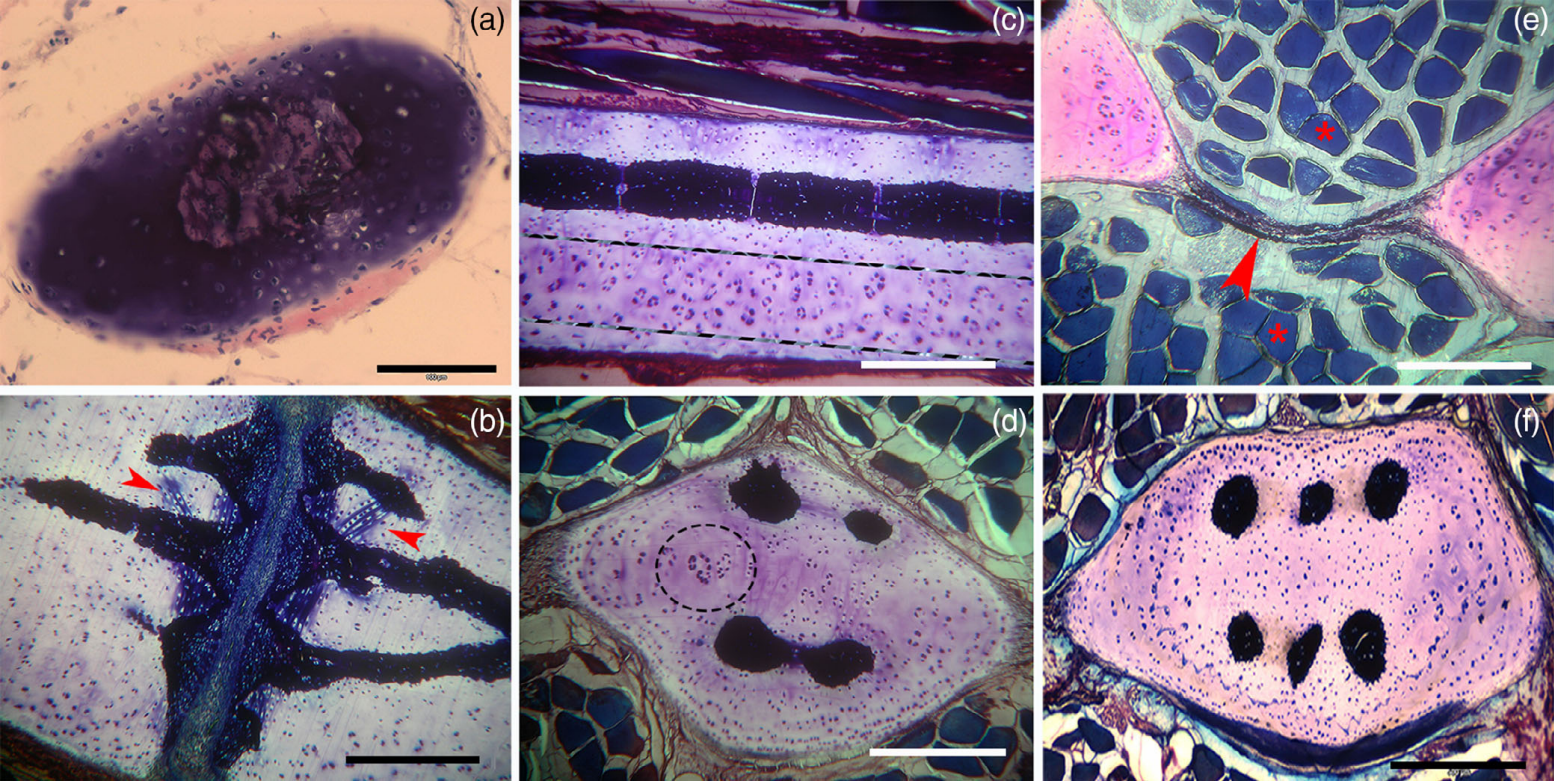
*Raja clavata* (Chondrichthyes). Undecalcified, resin‐embedded histology of wing‐fins and pelvic basipterygium radials, stained with methylene blue/acid fuchsine. (a) (200x) Mono‐columnar wing‐fin radial in transverse section showing the cartilage body of oval shape stained by methylene‐blue and the perichondrial layer by acid fuchsine. The calcified column shows a rounded shape in the central position. (b) (100x) Longitudinal section of the inter‐radials joint. The darker, blue‐stained zones correspond to the densely calcified matrix in the mass of uncalcified cartilage. Lighter methylene‐blue staining around aligned chondrocytes of increased volume (arrowheads) corresponds to a zone of impending calcification. (c) (100x) Longitudinal section of multi‐columnar basipterygium documenting a fully calcified column formed by cylindrical tiles. Below and parallel to the latter can be distinguished a zone of uncalcified cartilage (between dotted lines) characterized by a high density of duplicating chondrocytes and isogenic groups, suggesting a preparatory phase of cartilage matrix to mineral deposition and formation of a new column. (d) (100x) Transverse section of multi‐columnar basipterygium showing different stages of columns formation. The uncalcified area (within the dotted circle) shows a higher density of chondrocyte duplication corresponding to a zone of impending calcification (compared with that in the longitudinal plane of image c). (f) (100x) Transverse section of multi‐columnar basipterygium with the six completely calcified columns. (e) (100x) Transverse section of basipterygia showing the fibrous membrane (arrows) joining together two neighboring radials and separating the dorsal and ventral muscle fibers (asterisks). comment: order (f) after (e)

The central column, calcified cartilage of the wing‐fin radials, has a slender profile and a pattern of aligned, cylindrical tiles (Figure 6c). At the extremities, a calcified disk forms the base of the inter‐radial joints, which is sustained by short branches from the central calcified axis (Figure 6b). The radials show in transverse section an oval outline with the central column surrounded by a layer of uncalcified cartilage with a regular distribution of chondrocytes (index of low duplication rate and interstitial growth type of the tissue) (Figure 6a). Below the joint disks in the wing‐fins zone a, can be counted up to six branches supporting the disk (Figure 6f), while in the uncalcified cartilage around the radial columns can also be observed focal zones with high isogenic group density (index of tissue changes paving the way to incoming mineral deposition) (Figure 6c,d). The distribution of the latter (parallel to already calcified columns) supported the earlier hypothesis on the dynamics of mineral deposition and the concept of radials stiffening by adding new columns. Moreover, the transformation from cylindrical to polygonal “tiles” observed in the pelvic fins' longer and thicker radials fits with the stiffening mechanical hypothesis.

All the radials are bound transversally by a thick, uncalcified membrane which divides the dorsal from ventral muscles and works together with the stiff elements to modulate flexibility in the horizontal plane of the wing‐fin (Figure 6e).

**FIGURE 7:**
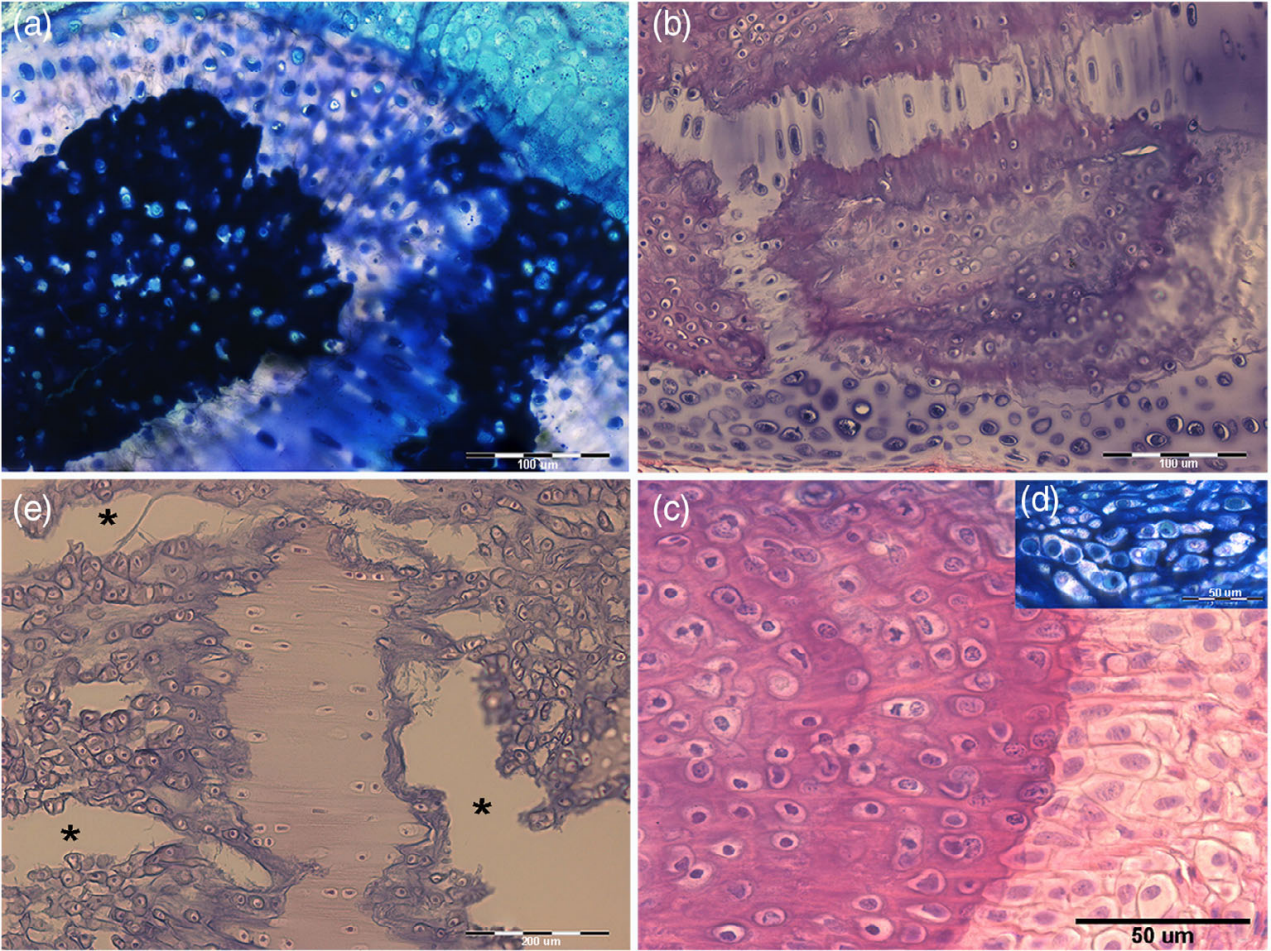
*Raja clavata* (Chondrichthyes). Undecalcified, resin‐embedded sections of pelvic basipterygium radials stained with methylene‐blue/acid fuchsine. (a) (200x) Transverse section of cartilage calcifying zone showing a mix composition of densely mineralized and impending calcification zones. An uncalcified matrix is evident in the top right corner. (b) (200x) Stripes of uncalcified cartilage separating calcified zones. The chondrocytes are increasing volume, and the cell duplications develop an irregular distribution of tensional stresses in this mixed tissue, as documented by the morphology of the stripe above (stretched in the plane of the section) and that of the stripe below (where tensional forces have a different direction). (c) (100x) Fissures (asterisks) produced by tensional stresses in the recent cartilage calcified matrix and associated with stretching the uncalcified cartilage. In the first, chondrocyte lacunae show a trend to form rows along the strain force direction. (d) (400x) Detail of the calcification border showing the large chondrocyte lacunae (with recent cell duplications in the top right insert) and chondrocytes hypertrophy in the layer of uncalcified cartilage on the right.

The calcification process in pterygia and radials does not differ from that reported in the endoskeleton segments (Pazzaglia et al., 2022), with a mixed distribution of calcified and uncalcified cartilage characterized by hypertrophic chondrocytes well demarcated from the lacunar border by a void space and frequently paired in the same lacuna (Figure 7a,c,d). The uncalcified sections histology provided evidence of the tensional stresses developing in this growing cartilage due to the mix of stiff (calcified) and elastic (uncalcified) tissue: the strips of the latter between the calcified zones showed stretched chondrocyte lacunae when the section plane was parallel to the tensional force vector or globular when perpendicular (Figure 7b). Another tension effect is documented in the same figure by the transverse micro‐fractures in the calcified cartilage and an alignment of chondrocytes parallel to the tensional vector (Figure 7e).

### 3.6 Scanning electron microscopy

**FIGURE 8:**
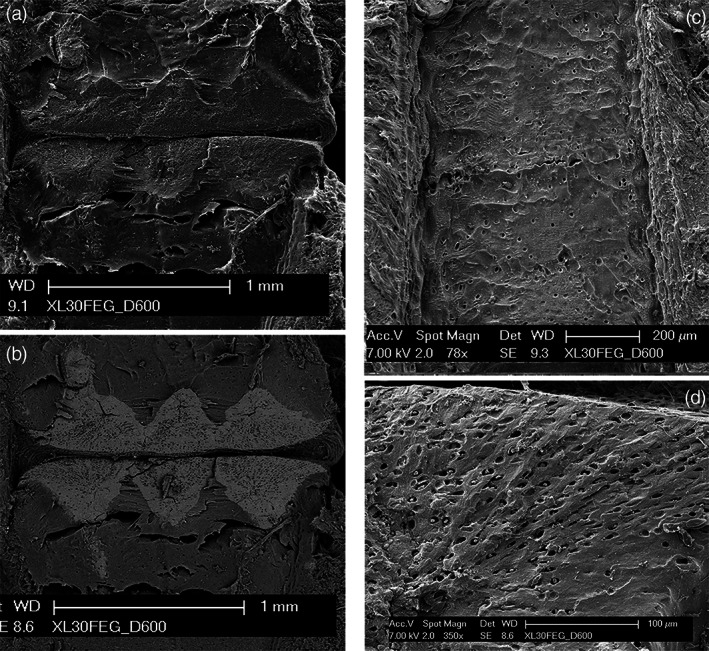
Raja *clavata* (Chondrichthyes) scanning electron microscopy images of wing‐fin radials. (a–b) (secondary electron imaging (SEI) and BSE mode of the inter‐radial joint zone) The BSE image shows the mineralized joint disks with a tesseral‐like layout, forming a compact and flat disk on both sides. The calcified cartilage has a higher density of chondrocytes lacunae than the neighboring, uncalcified cartilage. (c) (SEI of mono‐columnar radial longitudinally sectioned above the calcified central column). Regular, low‐density distribution of chondrocyte lacunae. On both the right and left sides are evident several perichondrial layers. (d) (SEI of a multi‐columnar basiptergium transversally sectioned). Zone of early calcification with a high density of chondrocyte lacunae aligned in rows.

Longitudinally fractured radials (at the level of inter‐radial amphiarthroses) were examined with SEM in both the secondary and back‐scattered modes, documenting three calcified cartilage blocks intersected by the fracture plane but leaving undetected the others forming the joint basal disk above or below the latter plane. They were characterized by a high density of chondrocyte lacunae (Figure 8a,b). Outwardly to the central column, the uncalcified cartilage showed a lower density of lacunae (Figure 8c), and a trend of alignment in rows of the latter could be observed in the transverse plane underneath the basal disk (Figure 8d).

## 4 DISCUSSION

The regular geometry of the Rajidae wing‐fin radials with a high number of elements provides a suitable model for a morphometric/morphological study of the combined cartilage ‐ mineralization pattern of growth in the class Chondrichthyes. Endochondral ossification in tetrapods provides the stiff substratum for bone apposition, while the mineralized structure of Chondrichthyes is formed inside the cartilage anlagen, remaining the definitive mechanical axis of the segment not undergoing further remodeling. Therefore, these fishes' fully cartilaginous skeletons must develop alternative strategies (different from teleosts and tetrapods) to accomplish their mechanical function throughout the life cycle. The comparative analysis of radials length in the target specimens 1 and 5 allowed to examine the wing‐find growth in relation to age, the youngest (110 g) and the oldest fish (1250 g), respectively, documenting a significant decrement of radials mean length among the inner, middle and outer zones of the wing‐fin central sector. The distribution of inter‐radial joints showed a regular layout of curved rows parallel to the propterygium and the metapterygium, which suggests that the radials' length growth in the whole wing was set in such a way to maintain the ordered alignment of the joints. In the anterior and posterior wing‐fins sectors, the joint alignment is also achieved by reducing the radial number in the ray. This confirms a controlled radial length growth adapted to the mechanical demand. Schaefer and Summers (2005) have reported in skates and rays “wings” a complex and phylogenetically diversified layout, including the calcification pattern of the wing‐fin segments. These authors further distinguished a “catenated” or “crustal” mineralized structure of radials, and it is interesting to underscore that the wing images of *Gymnura marmorata* and *Urobatis halleri* clearly showed a similar, ordered alignment of the inter‐radials joints parallel to the external outline of the wing, suggesting a specific phylogenetic character of the Rajidae family. These authors also reported inter‐species or intra‐individual variations of the mineralization pattern. In this study, shape differences between endoskeleton tesserae and radials cylindrical tiles were observed with a shape modulation in relation to the sectional area but without structural differences in the calcified cartilage. Maxwell et al. (2008) documented that the pectoral and pelvic fins appear as slightly lobed protrusions of the embryo body (fin buds) at stage 25 of the winter skate *Leucoraja ocellata*, with the pectoral bud forming two distinct lobes and the pelvic buds having become barely divided into anterior and posterior lobes at stage 28. The skeletal elements (rays and radials) of the wing‐fin were observed to begin as condensation of prechondrogenic mesenchyme later, undergoing segmentation at the joint sites (inter‐radial joints) with a developmental mechanisms pattern shared by fins with the limbs of other vertebrates (Cohn et al., 2002; Gillis et al., 2009; Hall, 2005, 2007). This mechanism explains how the number of rays in the wing becomes defined during embryonic development through hatching (Marconi et al., 2019), combining cartilage growth‐calcification throughout the fish life (Dean et al., 2009). This suggested that radial segmentation (Stern, 1990) occurred in the early developmental phase before calcification and that in the following growth period, chondrocytes proliferation and matrix calcification concurred to modulate stiffness/elasticity of the whole wing to the oscillatory, undulatory or mixed swim mode in each batoid species (Blevins & Lauder, 2012; Di Santo et al., 2017; Heine, 1992; Klausewitz, 1964; Rosenberger, 2001).

To the best of our knowledge, the microscopic trans‐illumination combined with the heat deproteination technique has not been so far applied to morphological and morphometric studies of cartilage calcification in the Rajidae wing‐fins. Our results have provided the following observations: 1) microstructure of the wing cylindrical tiles like that of the endoskeletal tesserae (Maisey et al., 2020; Seidel et al., 2016, 2017), the difference lying only in shape; 2) assemblage in columns of mono‐, bi‐, and multi‐columnar morphotypes (in the latter with a gradual transition to a peripheral crustal pattern); 3) decrement of wing‐fin radials length from the basal line to the wing edge; 4) bifurcated tilel type connecting bi‐columnar or multi‐columnar radials. The combination of all these morphoanatomical traits displays a wing fins mechanical model with a diversified distribution of stiff and mobile segments which can be summarized as follows: 1) radials, whose stiffness is provided by the inner, calcified axes; 2) hinges with variable mobility such as the diarthrodial joints between pterygia ‐ girdles; b) amphiarthroses between pterygia ‐ 1st line radials and inter‐radials joints; 3) the transverse inter‐radials ligaments, forming the flat membrane wide‐ranging to the whole wing‐fin surface (Pazzaglia et al., 2022). A further factor contributing to the variable flexibility of the wing zones was the ray duplication at ≈2/3rd of wing breadth, doubling the number of stiff segments in the external sector but with thinner columns. This anatomical model fits well with the three‐dimensional kinematics of the wing‐fin surface suggested by Blevins and Lauder (2012) and Di Santo et al. (2017).

The Rajidae cartilage growth of both wing‐fins and pelvic girdle metapterygia share similar patterns with the tetrapod's metaphyseal growth plate, such as chondrocytes duplication, volume increase and steering in rows where the intercellular matrix mineralization plays a driving role in cells orientation. Particularly in tetrapods' growth plate, this occurs between the calcified inter‐columnar septa, which orient the duplicating chondrocytes along the long bone axis, an action which is strengthened externally by the perichondral bone bark (Dodds, 1930; Pazzaglia et al., 2017a; 2017b; 2018; Pratt, 1959; Shapiro et al., 1977). This structural layout is combined with increased chondrocyte volume (hypertrophy) and mineral deposition in the inter‐columnar cartilage. The latter forms the substratum for osteoblasts apposition, followed by remodeling of the mixed cartilage/osteoid calcified tissue (Pazzaglia et al., 2019; 2020). With the due differences, in Rajidae wing‐fin radials, longitudinal rows of chondrocytes have been observed in the cylindrical tiles, and the same pattern (but with a radial layout) is also present in the polygonal tesserae of the elasmobranchs crustal calcification (Maisey et al., 2020; Seidel et al., 2016, 2017). The main difference between the elasmobranch's calcifying cartilage is that the alignment of chondrocytes is less regular, and their size increase is not so much developed as that of the metaphyseal growth plate. Chondrocyte hypertrophy (or better cytoplasmic swelling) has been hypothesized to represent a mechanism of Ca and PO_4_ions concentration through the passage of water from the intercellular matrix into the cell (determined by osmotic equilibrium), resulting in calcium‐phosphate deposits in the matrix (Maroudas, 1976; Maroudas & Schneiderman, 1987; Pazzaglia et al., 2018, 2020). In the Rajidae calcifying cartilage the chondrocyte volume increases; they appear more densely packed and are surrounded by less intercellular matrix than the not‐calcifying zones (Pazzaglia et al., 2022). Therefore, no definitive conclusions can be drawn at present by comparison of these two calcification patterns, which certainly deserve more in‐depth comparative studies.

The ultrastructural and developmental features of the tessellated endoskeleton of elasmobranchs have been extensively studied using synchrotron high‐resolution 3‐D techniques revealing in the course of development a complex lamellar, high‐mineral density layout with structures radiating outward, like spokes on a wheel, from the center of each tesserae to the contact with the neighbors (Seidel et al., 2016, 2021). Our study carried out with heat deproteination at 400°C confirms the already reported morphology of the tesserae mineral phase and further documents in the cylinder‐shaped radial tiles an orientation parallel to the major axis of column subunits. Seidel et al. (2017) described in shark and ray calcified cartilage endophytic masses as an aberrant type of mineralization with a strikingly different morphology compared to the tesserae and the other calcified tissues of elasmobranchs. These authors interpreted the latter findings as a possible damage response of the tissue. In this study, heat‐deproteinated morphology of radials cylindrical tesserae has shown similar, compact mineral deposits externally to and with a different texture of the inner core of calcified cartilage, suggesting two distinct phases of the mineral deposition process.

To the best of our knowledge, few data have been so far provided on the cells and organic phase morphology in the calcified cartilage of elasmobranchs (Kemp & Westrin, 1979) that can indeed be obtained by the cutting‐grinding technique in resin‐embedded, undecalcified specimens. In this study, the latter technique showed a mixed tissue of calcified and uncalcified cartilage and evidence of the strain forces on the tissue: the more elastic uncalcified matrix (interposed between calcified zones) showing elongated chondrocyte lacunae along the applied force line. At the same time, fissures were produced in the neighboring calcified zones. It can be emphasized that these fissures (micro‐fractures) are not artifacts produced by a microtome blade because, in the present investigation, the slices have been made with cutting‐grinding processing. The fissures were observed in zones of high cell density with chondrocytes of large volume and a calcified intercellular matrix. Marconi et al. (2019) have reported similar chondrocyte features in the metapterygium of adult skate *Leucoraya erinacea* with the definition “terminally differentiated in non‐hypertrophic state chondrocytes”. It can also be emphasized that the mass of the calcified matrix between these cells is scarce and less compact than that of the metaphyseal growth plate of tetrapods. In the same study, experimental cartilage injuries were produced by removing small wedges of tissue from the metapterygium, further providing evidence of a reparative chondrogenesis capacity. The “non‐hypertrophic state of chondrocytes” does not exclude the mineral deposition process based on H_2_O recall into the cell and concentration of Ca and PO_4_ ions in the intercellular matrix as suggested for the growth plate cartilage (Pazzaglia et al., 2020). However, the different patterns of interstitial fluids diffusion in the two models could also have a role in the nucleation and CaPO_4_ salts deposition into the collagen matrix network. The X‐ray imaging and the microscopic observations of heat‐deproteinated wing‐fins specimens provided so far unreported data on morphology and calcification in the radials tiles showing an evident correlation with the mechanics of the wing‐fins.

To characterize and discriminate the taxa of the Mediterranean Rajidae, identification keys are currently used based on external and internal morphoanatomical traits (Serena et al., 2010), but doubtful cases can be occasionally discussed. Additional diagnostic morphotraits provided by X‐ray imaging and heat deproteination of the calcified skeleton could be useful to complete the classical morphological data and to solve doubtful taxonomic cases. However, how the mineral deposition in the radial columns and the crustal‐like pattern in the endoskeleton segments of the pelvic basipterygia and of the longer and thicker radials remains unresolved. Assuming that the chondrocyte metabolic activity regulates the mineral deposition in the peri‐cellular matrix, it should not be considered casual the changes in the chondrocyte's morphology and the organization of the peri‐cellular matrix in the impending calcification zone.

## AUTHOR CONTRIBUTIONS

**Ugo E. Pazzaglia:** Conceptualization; data curation; investigation; methodology; supervision; writing – original draft; writing – review and editing. **Marcella Reguzzoni:** Conceptualization; funding acquisition; investigation; methodology; supervision; writing – original draft; writing – review and editing. **Renata Manconi:** Data curation; methodology; resources; validation; writing – review and editing. **Piero Antonio Zecca:** Data curation; funding acquisition; software; visualization. **Guido Zarattini:** Data curation; investigation; validation; visualization. **Monica Campagnolo:** Investigation; methodology; visualization. **Mario Raspanti:** Funding acquisition; project administration; validation.

## CONFLICT OF INTEREST

The authors declare that they have no competing financial interests or personal relationships that could have influenced the work reported in this paper.

## Data Availability

The data that support the findings of this study are available from the corresponding authors upon reasonable request.
